# The Use of High-Protein Preparations in Ice Cream Production

**DOI:** 10.3390/foods14030345

**Published:** 2025-01-21

**Authors:** Katarzyna Kiełczewska, Michał Smoczyński, Marta Gutkowska

**Affiliations:** Dairy Science and Quality Management, Warmia and Mazury University, Oczapowskiego 7, 10-719 Olsztyn, Poland; kaka@uwm.edu.pl (K.K.); marta.gutkowska98@gmail.com (M.G.)

**Keywords:** ice cream mix, ice cream, high-protein preparations, rheology, colour, sensory attributes

## Abstract

The aim of this study was to evaluate the applicability of high-protein preparations in the production of ice cream. Ice cream for the experiment was produced with the addition of the following high-protein preparations: micellar casein concentrate (CN) obtained from skimmed milk, buttermilk protein concentrate (BMP), whey protein concentrate (WPC) with 80% protein content, and skimmed milk powder (SMP) as the control sample. The ice cream mix (composition, colour, and consistency index) and the ice cream (overrun, melting rate, hardness, and sensory attributes) were analysed in this study. The addition of high-protein preparations increased the protein content of the ice cream mix, thus modifying selected properties of the mix and the produced ice cream. Mixes fortified with high-protein preparations were characterised by a higher consistency index (maximum values for WPC) and larger particle size (maximum values for CN) than those of the control sample. The whiteness index was lower in high-protein ice cream mixes than in the control sample. Depending on the type of preparation added to the ice cream mix, the resulting ice cream differed in hardness (hardness was highest in samples containing WPC, 276.54 N), overrun (lowest in samples containing WPC, 52.40%), and melting rate (lowest in samples containing BMP and highest in samples containing WPC, 0.24 g/min). High-protein preparations did not induce significant changes in ice cream palatability, except for ice cream fortified with WPC, which scored lower in the sensory analysis due to lower fluffiness, higher brittleness, and sour aroma and taste.

## 1. Introduction

Ice cream is one of the most popular desserts in the world. Cais-Sokolińska [1] defined ice cream as a pasteurised and frozen food product made from a fat emulsion, proteins, other ingredients, and additives. According to Kobyłko [2], ice cream is a complex multiphase system, where air cells are dispersed in an incompletely frozen continuous phase (water) and the remaining ingredients occur in the form of an emulsion (fat, stabilisers), suspensions, colloids (proteins), and solutions (sugar, salts). Ice cream is a source of fat-soluble vitamins (A, D, E, and K), B vitamins, highly available calcium, and other minerals, including phosphorus, magnesium, potassium, sodium, and zinc [3]. Ice cream also contains stabilisers and emulsifiers to ensure that the final product has the optimal consistency, structure, and overrun [2].

Contemporary consumers have a growing interest in the quality and nutritional value of food products. Food products belonging to the functional food segment are present in every sector of the food industry, including the dairy industry. According to Moughan [4], functional foods can be naturally functional or can be formulated by adding bioactive compounds or removing ingredients that suppress the bioactive properties of other compounds. Dairy products fortified with protein, including high-protein preparations, have attracted considerable interest in recent years. Protein is an essential macronutrient, and adequate dietary protein intake is necessary for optimal health [28). Milk proteins are high-quality proteins that have multifunctional properties, are easily digestible [4,5], and provide health benefits [6]. The functional properties of milk proteins also play an important role in food processing by improving the stability of emulsions, foams, and gels. Due to its amphiphilic character, casein is a surface-active compound with foaming and emulsifying properties [5]. Beta-casein is a particularly powerful foaming and emulsifying agent. According to Brodziak et al. [7], whey proteins are functional ingredients that are highly valued for their gelling properties, as well as their high-solubility, thickening, emulsifying, and foaming properties. Beta-lactoglobulin is the whey protein with the highest gelling capacity. According to Patel et al. [8], this whey protein fraction can be used in the production of yogurt, cream, confectionary, processed meats, and ready-made meals. Paglia et al. [9] observed that milk proteins are natural emulsifiers that are used in the production of clean label ice cream.

One of the trends in the ice-cream market is reducing the energy value by changing the recipe to reduce the sugar or fat content [1], by adding various polysaccharides [10], or by using milk substitutes such as whey [11]. The market of protein preparations that can be added to dairy-based frozen desserts, including ice cream, is interesting but underdeveloped. Due to their technological properties, high-protein preparations not only improve the nutritional value of ice cream but can also favourably influence its physicochemical and sensory characteristics.

Proteins can bind water; have hydrating, gelling, and thickening properties; and increase the viscosity of foods, which is why they can be used to stabilise phase systems and the consistency of ice cream mixes and ice cream. Therefore, the aim of this study was to determine the applicability of different high-protein preparations in the production of ice cream, based on an evaluation of the selected physicochemical and rheological properties of ice cream mixes, as well as the physical, rheological, and sensory properties of the ice cream produced from these mixes.

## 2. Materials and Methods

### 2.1. Materials

The experimental materials were skimmed milk and sweet cream separated from raw milk (Research and Education Station in Bałdy, University of Warmia and Mazury in Olsztyn); skimmed milk powder (SMP) (Mlekpol, Grajewo, Poland); whey protein concentrate (WPC) with 80% protein content (Superior Ltd., Olsztyn, Poland); micellar casein concentrate (CN)—retentate obtained from microfiltered skimmed milk (MF 50 °C, CP X3, 0.1 µm) and subjected to diafiltration (DF 50 °C, CP X3, 0.1 µm); and buttermilk protein concentrate (BMP)—retentate obtained from microfiltered buttermilk (MF 50 °C, CP X3, 0.1 µm). The obtained fractions were dried (Niro Atomiser, Soborg, Denmark) [12,13].

Ice cream mixes were prepared from skimmed milk, sweet cream, sugar, milk powder, or high-protein milk powder. The content of solids-not-fat, fat, and added sugar was standardised in the formulation of the mixes. These ingredients were combined in the following proportions: milk solids-not-fat—10%; milk fat—10%; sugar—10%; stabilising and emulsifying agent (Superior Ltd., Olsztyn, Poland)—0.3%. The ice cream mix was homogenised, pasteurised, aged, and frozen (Easyfreeze 2000/HT, Promag, Bologna, Italy). The obtained ice cream was placed in cups and hardened at a temperature of −20 ± 2 °C for 24 h. After hardening, ice cream was stored in a freezer (−15 °C) for around 10 days. The ice cream mix with the addition of SMP and the ice cream made from this mix were the control samples.

### 2.2. Methods

#### 2.2.1. Analysis of Ice Cream Mixes

##### Proximate Analysis

The chemical composition of ice cream mixes was determined with the FoodScan^TM^ Lab 78810 analytical instrument (Foss Analytical AB, Hillerød, Denmark).

##### Particle Size Analysis

Ice cream mixes were subjected to particle size analysis by laser diffraction using the Mastersizer 3000 particle size analyser with a Hydro EV disperser (Malvern Instruments, Worcestershire, UK). The results were expressed as the Sauter mean diameter (d_32_) of particles (volume–surface mean diameter, d_32_ = Σ Sd_i_^3^ n_i_/Sd_i_^2^ n_i_) and the De Brouckere mean diameter (d_43_) (volume-weighted mean diameter, d_43_ = Σ Sd_i_^4^ n_i_/Sd_i_^3^ n_i_), where n_i_ is the number of particles with diameter d_i_.

##### Colour Analysis

The colour of ice cream mixes was analysed with the CM-3500d spectrophotometer (Konica Minolta Sensing, Inc. Osaka, Japan) in the CIE LAB colour space, and it was expressed by components L* (L* = 0 for black, L* = 100 for white colour), a* (−a* = greenness, +a* = redness), and b* (−b* = blueness, +b* = yellowness). The values of L*, a*, and b* were used to calculate chroma/colour saturation C* and the whiteness index WI according to the following formulas [14]:

C* = (a*^2^ + b*^2^)^1/2^

WI = 100 − [(100 − L*)^2^ + (a*)^2^ + (b*)^2^]^1/2^

##### Rheological Properties

Shear stress was measured using a Rheolab QC rotational rheometer (Anton Paar Gmbh, Graz, Austria) with CC27-SN20703 cylinder geometry, at a temperature of 20 °C, and a shear rate of 1–100 s^−1^. The results were used.

Moreover, d was used to plot flow curves describing the relationship between shear stress τ and shear rate γ. Rheological properties were fitted to the Ostwald de Walle model, and mathematical models were used to calculate the consistency index as a measure of apparent viscosity [15].

τ = k*γ^n^ (1)

where τ—shear stress, Pa; k—consistency index, Pa*s^n^; γ—shear rate, s^−1^; n—flow behaviour index.

#### 2.2.2. Ice Cream Analysis

##### Melting Rate

The mass of a melted 100 g ice cream sample was determined every 10 min over a period of 1 h. The percentage of melted mass relative to the initial mass was calculated, and the melting rate of ice cream was determined using the following formula [4]):

$$Melting\;rate\;(\frac{g}{60}min)=\frac{Mass\;of\;melted\;ice\;cream\;(g)}{Time\;(min)}$$

##### Hardness

The hardness of ice cream samples was determined with a texture analyser (TA-XT2i, Stable Micro Systems, Godalming, UK). To minimise the temperature difference between the ice cream sample and the probe, the probe was kept in a freezer at a temperature of −20 ± 2 °C for 30 min before the experiment.

##### Overrun

The same volumes of the ice cream mix and ice cream (100 mL) were weighed. Overrun was calculated using the following formula [16]):

$$Overrun(\%)=\frac{W_{1}-W_{2}}{W_{2}}\times 100$$

where W_1_—mass of the ice cream mix; W_2_—mass of ice cream.

### 2.3. Sensory Analysis

The sensory attributes of ice cream samples were analysed in a sensory laboratory with the use of a standard profiling method [17]. The evaluation was carried out by a panel of eight suitably trained persons with the appropriate sensory sensitivity, according to the ISO Standard method [18]. Each sample was coded with a random three-digit number and served in 100 mL transparent propylene cups. During the sensory analysis, every panellist received an attribute card and a score card. The cards contained a total of 20 attributes. Sensory attributes were assessed on a 5-point descriptive scale, where 1 point denoted the absence of a given attribute, and 5 points denoted high intensity of the examined attribute.

### 2.4. Statistical Analysis

The results of the sensory analysis were processed by one-way ANOVA, and the presence of significant differences between variables was determined by Fisher’s LSD test. All calculations were performed using the Microsoft Office Excel 2013 package at a significance level of 0.05.

## 3. Results and Discussion

### Ice Cream Mix

The ice cream mix with the addition of CN was characterised by the highest protein content. No significant (*p* = 0.05) differences in protein content were observed between ice cream mixes fortified with WPC and BMP. The content of total solids and fat in ice cream mixes was consistent with the recipe (Table 1). Other authors also reported an increase in the protein content of ice cream mixes due to the addition of whey protein-based formulations [8,19,20]. The increased protein content of ice cream not only improves its nutritional value but may also affect its structural properties [21]. Hossain et al. [20] observed that protein content was bound by a strong positive correlation with the melting rate and a negative correlation with viscosity.

Ice cream mixes were characterised by a shear rate-dependent flow curve conforming with the Ostwald de Waele power equation. Ice cream mixes presented the features of a shear-thinning non-Newtonian fluid. In this case, its viscosity was inversely proportional to the shear-thinning shear rate (n < 1) (Table 2). High-protein preparations modified the rheological characteristics of ice cream mixes, and the observed changes were determined by the type of preparation used in the production process. Consistency index values of mixes fortified with CN, BMP, and WPC were three, two, and eight times higher, respectively, as compared to that of the control sample (Table 2). High-protein preparations increased the consistency index of ice cream mixes by promoting the formation of large protein networks that absorb water and exhibit thickening and gelling properties [22]. In other studies, the addition of WPC also increased the viscosity of ice cream mixes [21,22]. Ruger et al. [23] found that ice cream consistency improved with an increase in the viscosity of the ice cream mix. These observations indicate that milk proteins exert a positive influence on the rheological properties of ice cream mixes.

The high-consistency-index values of the ice cream mix containing high-protein preparations were confirmed by a significantly larger particle size relative to that of the remaining samples, especially in the case of the application of CN (Table 3).

Ice cream mixes with the addition of high-protein preparations were characterised by lower values of parameter L* than those of the control sample (Table 4). The mix containing WPC had the darkest colour. Akalın et al. [19] and Roland et al. [24] also reported lower values of L* in ice cream produced with the addition of high-protein preparations than in the control samples. In contrast, Kiełczewska et al. [25] noted higher values of L* in non-fermented milks enriched with high-protein preparations. The cited authors found that the value of parameter L* increased with a rise in casein content. These discrepancies may be due to the fact that ice cream mixes have a high milk fat content. According to Hossain et al. [20], the colour lightness of ice cream increases significantly with increasing milk fat content, which was not observed in the present study.

All samples were characterised by negative values of parameter a* and positive values of parameter b*. Similar observations were made in studies where ice cream was produced with the addition of a milk protein concentrate [24] and buttermilk powder [26,27]. In the current study, the values of colour parameters a* and b* differed across samples (Table 4). Mixes fortified with high-protein preparations were characterised by higher values of parameter a*. The values of parameter b* varied depending on the type of high-protein preparation, and they were higher in mixes containing BMP and WPC and lower in mixes containing CN relative to the control sample. The differences in the colour parameters of ice cream mixes fortified with different high-protein preparations were also reflected in the values of parameter C*, which were lowest in the mix with the addition of CN. In mixes containing the remaining high-protein preparations, chroma/colour saturation was higher than in the control sample. High-protein preparations decreased the value of the WI. The value of parameter WI was lowest in the ice cream mix containing WPC, probably due to the absence of the colloidal particles of the casein and calcium complex in the WPC80 preparation, which are responsible for the white colour of milk [28].

The modification of the composition of ice cream mixes through the addition of high-protein preparations affected the properties of the ice cream produced from these mixes. The replacement of SMP with WPC led to a more than two-fold increase ice cream hardness and decreased overrun by around a third. The remaining samples did not differ in hardness. Due to higher overrun, these samples were characterised by lower hardness than those produced from the mix fortified with WPC (Table 5).

An increase in the hardness of ice cream (Table 5) was observed with the increase in the consistency index values of ice cream mixes (Table 2). This relationship was also described by Akalın et al. [19] and Paglia et al. [9] in their research. Fluffier ice cream can be less resistant to penetration in the hardness test, which implies that samples with higher overrun are characterised by a less compact structure and lower hardness [26,29]. Roy et al. [30] analysed ice cream produced with the addition of whey protein isolate (WPI) and concluded that the strong network formed by β-lactoglobulin could have contributed to increased hardness in samples containing WPI. In the work of Akalın et al. [19] and Danesh et al. [31], ice cream hardness also increased after the addition of a whey protein preparation. Harder ice cream was produced from mixes where particle size distribution was influenced by the formation of milk protein networks. Based on this observation, ice cream cohesiveness can be indirectly defined as the strength of internal bonds constituting the texture of the product [21].

High overrun in ice cream containing CN and BMP could be attributed to increased casein content which promotes the formation of air cells and foam [32]. In turn, ice cream with the addition of WPC was characterised by lower overrun than that of the control sample, which corroborates the findings of Khillari et al. [32] and Gurskiy and Tvorogova [22]. Lower overrun could have resulted from the destabilisation of air cells in successive stages of freezing [29] or lower stability of the foam produced by WPC. In addition, overrun is difficult to control in the production of small batches of ice cream [8].

In ice cream, overrun is an important quality parameter which is defined as the percentage increase in volume resulting from the amount of air incorporated into the product during freezing [29]. The foam produced during this process is stabilised with surface-active agents, and it is collected along the phase boundary [21]. The amount of introduced air affects sensory properties, consistency, and melting rate [27].

The melting rate was lowest in ice cream with the addition of BMP and highest in ice cream with the addition of WPC (Table 5). According to El-Zeini et al. [21], ice cream with a high content of WPC tends to melt more rapidly. In the cited study, the melting rate decreased when WPC content was increased up to 3%, but ice cream became more susceptible to melting with a further increase in WPC content. Similar observations were made by Khillari et al. [32].

The sensory analysis revealed that the examined ice creams did not differ significantly in appearance, did not exhibit atypical discolouration or phase separation, and were characterised by a uniform creamy-white colour (Table 6). The panellists did not identify significant differences in most aroma descriptors. The samples had a typical, milky and moderately sweet aroma. However, ice cream containing WPC had a more intense sour aroma than that of other ice creams. In contrast, Danesh et al. [31] found that the addition of a whey protein preparation did not induce noticeable changes in the aroma of ice cream.

The analysed ice creams did not differ in textural properties, except for fluffiness and brittleness. Ice cream with the addition of WPC received the lowest scores for fluffiness and the highest scores for brittleness although no differences in hardness were found (Table 6). In terms of fluffiness and brittleness, the results of the sensory analysis are consistent with the previously determined differences in hardness and overrun (Table 5). On the contrary, El-Zeini et al. [21] reported that the addition of WPC had no influence on ice cream consistency. The addition of milk proteins, which have gelling properties, can contribute to a desirable mouthfeel in ice cream [33], but the examined samples did not differ significantly in the descriptors of this parameter. The panellists did not observe any differences in the melting rate of ice cream samples although such variations had been noted in previous analyses. Ice crystals were weakly detectable in ice cream. The high water-binding capacity of milk proteins contributes to the smooth texture of ice cream by preventing the formation of large ice crystals and [21].

Taste is a very important attribute determining the quality of ice cream. The examined ice cream was produced without any flavourings, and the milky taste was most distinctive in all samples. According to the panellists, the sour taste was most intense in ice cream containing WPC. In the remaining samples, no significant differences in taste were determined at *p* ≤ 0.05. The sensory analysis also revealed a relatively strong milk powder taste, which is undesirable in ice cream [24]. Similar results were reported by El-Zeini et al. [21] who found that ice cream with too high WPC addition scored lower for taste.

## 4. Conclusions

The addition of high-protein preparations, especially WPC, increased the consistency index of ice cream mixes. Ice cream mixes fortified with high-protein preparations were characterised by lower values of L* and a lower contribution of greenness (a*), whereas mixes fortified with WPC and BMP were also characterised by a higher contribution of yellowness (b*) and higher chroma/colour saturation (C*) than those of the control sample. The addition of high-protein preparations affected the physical and textural properties of ice cream by increasing overrun (WPC and CN), decreasing the melting rate (BMP), and increasing hardness (WPC). Both CN and BMP improved the quality attributes of ice cream (melting rate and overrun), compared to those of the commercial WPC. The addition of high-protein preparations had no significant effect on the sensory attributes of ice cream. Only WPC increased the intensity of the sour aroma and taste, decreased fluffiness, and increased brittleness of ice cream. High-protein preparations offer an interesting alternative to milk powder in ice cream production and can improve the nutritional value of the final product.

## Figures and Tables

**Table 1:** Composition of ice cream mixes fortified with high-protein preparations.

High-Protein Preparation	Total Solids, %	Fat, %	Protein, %
SMP	29.99 ± 0.05	10.09 ± 0.04	2.98 ± 0.06 ^a^
CN	30.01 ± 0.16	10.13 ± 0.02	5.87 ± 0.11 ^d^
BMP	29.98 ± 0.08	10.49 ± 0.04	4.97 ± 0.09 ^b^
WPC	29.99 ± 0.11	9.97 ± 0.02	5.17 ± 0.12 ^c^

Values are means ± SD (n = 3). Values with different superscripts in columns differ significantly at *p* < 0.05. SMP—skimmed milk powder; CN—casein; BMP—buttermilk protein; WPC—whey protein concentrate.

**Table 2:** Rheological properties of ice cream mixes fortified with high-protein preparations.

High-Protein Preparation	Consistency Index, Pa s^n^	Flow Rate	Determination Coefficient R^2^
SMP	0.0528 ± 0.0017 ^a^	0.7186 ± 0.01 ^c^	0.997
CN	0.1686 ± 0.0022 ^c^	0.7046 ± 0.65 ^c^	0.997
BMP	0.1135 ± 0.0011 ^b^	0.6071 ± 0.01 ^b^	0.995
WPC	0.4717 ± 0.0017 ^d^	0.3282 ± 0.05 ^a^	0.995

Values are means ± SD (n = 3). Values with different superscripts in columns differ significantly at *p* < 0.05. SMP—skimmed milk powder; CN—casein; BMP—buttermilk protein; WPC—whey protein concentrate.

**Table 3:** Particle size in ice cream mixes fortified with high-protein preparations.

High-Protein Preparation	D_32_, μm	D_43_, μm
SMP	0.10 ± 0.01 ^a^	1.95 ± 0.29 ^a^
CN	15.22 ± 0.65 ^b^	30.20 ± 0.61 ^b^
BMP	0.18 ± 0.01 ^a^	6.13 ± 0.14 ^a^
WPC	0.13 ± 0.01 ^a^	2.61 ± 0.68 ^a^

Values are means ± SD (n = 3). Values with different superscripts in columns differ significantly at *p* < 0.05. SMP—skimmed milk powder; CN—casein; BMP—buttermilk protein; WPC—whey protein concentrate.

**Table 4:** Colour parameters of ice cream mixes fortified with high-protein preparations.

High-Protein Preparation	L*	a*	b*	C*	WI
SMP	91.38 ± 0.54 ^c^	−3.34 ± 0.06 ^d^	13.76 ± 0.48 ^b^	13.99 ± 0.20 ^b^	90.80 ± 0.48 ^a^
CN	90.13 ± 0.14 ^b^	−2.81 ± 0.16 ^b^	12.94 ± 0.23 ^a^	13.24 ± 0.25 ^a^	89.63 ± 0.13 ^b^
BMP	90.12 ± 0.08 ^b^	−3.11 ± 0.10 ^c^	14.10 ± 0.15 ^c^	14.44 ± 0.12 ^c^	89.58 ± 0.28 ^b^
WPC	88.65 ± 0.57 ^a^	−2.12 ± 0.25 ^a^	14.74 ± 0.11 ^d^	14.89 ± 0.14 ^d^	88.11 ± 0.56 ^c^

Values are means ± SD (n = 3). Values with different superscripts in columns differ significantly at *p* < 0.05. C*—chroma/colour saturation; WI—whiteness index; SMP—skimmed milk powder; CN—casein; BMP—buttermilk protein; WPC—whey protein concentrate.

**Table 5:** Properties of ice cream fortified with high-protein preparations.

High-Protein Preparation	Hardness, N	Overrun, %	Melting Rate, g/min
SMP	118.16 ± 33.02 ^a^	75.68 ± 2.57 ^b^	0.10 ± 0.03 ^b^
CN	138.43 ± 14.95 ^a^	79.66 ± 0.62 ^c^	0.12 ± 0.01 ^c^
BMP	113.15 ± 37.64 ^a^	82.39 ± 2.84 ^c^	0.05 ± 0.01 ^a^
WPC	276.54 ± 18.05 ^b^	52.40 ± 2.45 ^a^	0.24 ± 0.06 ^d^

Values are means ± SD (n = 3). Values with different superscripts in columns differ significantly at *p* < 0.05. SMP—skimmed milk powder; CN—casein; BMP—buttermilk protein; WPC—whey protein concentrate.

**Table 6:** Sensory attributes of ice cream fortified with high-protein preparations.

High-Protein Preparation		SMP	CN	BMP	WPC	*p*-Value
Appearance	Creamy-white colour	3.7	3.6	3.9	3.9	>0.05
	Colour uniformity	5.0	5.0	5.0	5.0	>0.05
	Phase separation	1.0	1.0	1.0	1.0	>0.05
Aroma	Milky	4.0	4.1	4.0	4.1	>0.05
	Sweet	3.6	3.8	3.9	3.9	>0.05
	Sour	1.0 ^a^	1.0 ^a^	1.1 ^a^	2.1 ^b^	0.0013
	Atypical	1.1	1.4	1.3	1.3	>0.05
Consistency	Hardness	3.8	3.6	3.8	3.9	>0.05
	Uniformity	4.8	4.6	4.8	4.7	>0.05
	Smoothness	4.8	4.8	4.8	4.5	>0.05
	Lumpiness	1.0	1.0	1.0	1.0	>0.05
	Fluffiness	4.0 ^b^	4.0 ^b^	4.0 ^b^	3.6 ^a^	0.002
	Melting	3.6	3.8	3.8	4.0	>0.05
	Brittleness	1.8 ^a^	1.6 ^a^	1.6 ^a^	2.7 ^b^	0.0014
	Ice crystallisation	1.13	1.25	1.25	1.25	>0.05
Taste	Milky	4.0	4.1	4.0	4.1	>0.05
	Sweet	3.9	3.8	3.7	4.0	>0.05
	Sour	1.0 ^a^	1.2 ^a^	1.1 ^a^	2.5 ^b^	0.001
	Cooked	1.6	1.6	1.5	1.6	>0.05
	Atypical	1.0	1.2	1.1	1.3	>0.05

Values are means (n = 8). Values with different superscripts in rows differ significantly at *p* < 0.05. SMP—skimmed milk powder; CN—casein; BMP—buttermilk protein; WPC—whey protein concentrate.

## Data Availability

The original contributions presented in the study are included in the article, further inquiries can be directed to the corresponding author.
